# Unveiling water dynamics in fuel cells from time-resolved tomographic microscopy data

**DOI:** 10.1038/s41598-020-73036-w

**Published:** 2020-10-02

**Authors:** Minna Bührer, Hong Xu, Jens Eller, Jan Sijbers, Marco Stampanoni, Federica Marone

**Affiliations:** 1 Swiss Light Source, Paul Scherrer Institut, Forschungsstrasse 111, 5232 Villigen, Aargau Switzerland; 2 Institute for Biomedical Engineering, University and ETH Zürich, 8092 Zürich, Zürich Switzerland; 3 Electrochemistry Laboratory, Paul Scherrer Institut, Forschungsstrasse 111, 5232 Villigen, Aargau Switzerland; 4 imec-Vision Lab, Department of Physics, University of Antwerp, Universiteitsplein 1, 2610 Antwerpen, Antwerpen Belgium

**Keywords:** Fuel cells, X-rays, Phase-contrast microscopy, Computational science, Software, Imaging techniques

## Abstract

X-ray dynamic tomographic microscopy offers new opportunities in the volumetric investigation of dynamic processes. Due to data complexity and their sheer amount, extraction of comprehensive quantitative information remains challenging due to the intensive manual interaction required. Particularly for dynamic investigations, these intensive manual requirements significantly extend the total data post-processing time, limiting possible dynamic analysis realistically to a few samples and time steps, hindering full exploitation of the new capabilities offered at dedicated time-resolved X-ray tomographic stations. In this paper, a fully automatized iterative tomographic reconstruction pipeline (rSIRT-PWC-DIFF) designed to reconstruct and segment dynamic processes within a static matrix is presented. The proposed algorithm includes automatic dynamic feature separation through difference sinograms, a virtual sinogram step for interior tomography datasets, time-regularization extended to small sub-regions for increased robustness and an automatic stopping criterion. We demonstrate the advantages of our approach on dynamic fuel cell data, for which the current data post-processing pipeline heavily relies on manual labor. The proposed approach reduces the post-processing time by at least a factor of 4 on limited computational resources. Full independence from manual interaction additionally allows straightforward up-scaling to efficiently process larger data, extensively boosting the possibilities in future dynamic X-ray tomographic investigations.

## Introduction

X-ray tomography is a common imaging technique used to investigate the interior of samples in a non-invasive manner. Through tomographic microscopy, inner structures of an object can be reconstructed from a set of 2D radiographs into a 3D volume. Dynamic computed tomography has extended static investigations to time-evolving processes enabling to follow, for example, bubble growth in basaltic foams^1^, cracking during in situ tensile tests^2^, investigate in vivo the muscular mechanics of a blow-fly^3^ in 4D, observe in situ percolation threshold in multiphase magma analogues^4^ and follow evolution of a metal foam at a record-breaking time-resolution of 208 tomograms per second^5^. As the ideal scan time for each dynamic sequence is bounded by the speed of the evolving process in question, both exposure time and angular sampling frequency are typically severely limited. When aiming to follow a rapidly evolving system in real-time, this results in tomograms that are highly noisy and degraded by undersampling artefacts. Extracting the evolving dynamics from these datasets requires typically data post-processing pipelines consisting of data reconstruction, registration, filtering and segmentation steps. Usually each single step requires parameter tuning and manual optimization. These intensive manual requirements significantly extend the total data post-processing time well beyond the actual computational time. Moreover, the parameters optimized for one dataset do typically not generalize well. Post-processing large amounts of data for multiple samples and different experimental conditions as often required in investigations of real-life processes becomes an insurmountable task. This reality leads either to cutting-edge experiments for which the acquired data are only partially analyzed, or to experimental plans limited to just a small range of the full parameter space. In both cases, the final results only marginally take advantage of the full potential of new dedicated time-resolved X-ray tomographic stations.

Dynamic sub-second X-ray tomographic microscopy is an invaluable technique to investigate liquid water dynamics in polymer electrolyte fuel cells (PEFCs) during cell operation. Fuel cells are a promising technology to decarbonize the mobility sector. Suboptimal water management during cell operation is though one of the major limiting factors for increasing its performance at high current density operations. To investigate the water dynamics during transient operation through X-ray tomography, it is necessary to push the scan time of the collected tomograms to the sub-second regime^6,7^, leading to highly restricted angular sampling frequency and exposure time. Standard analytical reconstruction algorithms, such as filtered back-projection (FBP)^8^ and Gridrec^9,10^, consider each time frame of a dynamic sequence independently without exploiting the additional information across the time sequence. In this way, they have limited ability to cope with undersampled and noisy datasets, leading to challenges in further data analysis and post-processing and so, increasing the necessary data analysis time significantly. As the gray level value of the reconstructed liquid water is very close to the values of the static structures and noise, isolating water signals directly from the reconstructed highly noisy dynamic volumes is not possible. Instead, a high-quality *post* *operando* dataset of the fuel cell in a dry state has to be acquired and aligned with the dry structures of each reconstructed dynamic time frame, each suffering from low signal-to-noise ratio (SNR). The aligned reconstructions are subtracted to obtain difference images containing only liquid water features and background noise, and further post processed by a dedicated ad hoc pipeline^11,12^. Misalignments between the static and dynamic reconstructions lead to artefacts in the difference images, resulting in an increased risk of misidentified water features. In addition, due to varying imaging protocols between different experiments, full automatization of the segmentation and alignment pipeline is currently not possible as the post processing parameters are strongly dependent on the experimental protocols and therefore need to be individually adjusted.

Several research activities have been on-going to exploit the information available in the time-domain within the reconstruction to improve the spatial resolution of the reconstructed volumes and so to push the temporal resolution of the experiment even further. Ruhlandt et al*.*^13^ applied optical flow analysis to derive a motion model from the direct analytical reconstructions. The resulting model was then used to adjust the back-projection geometry of the analytical reconstructions and perform the final reconstructions on dynamically curved paths to overcome artefacts caused by the sample evolving faster than the temporal resolution of the experiment. While the motion artefacts were shown to be suppressed through the proposed approach, for highly noisy datasets additional care might still be required to reduce the noise level of the analytical reconstructions for further image post-processing.

Iterative reconstruction algorithms have been developed to overcome reconstruction quality limitations of analytical methods when noisy and undersampled datasets, typical in real experiments, are present. To consider a time-sequence of data within iterative reconstruction, Kazantsev et al*.*^14,15^ proposed a spatial–temporal patch-based regularization technique to perform non-local image denoising by extracting information across the complete time-domain through weighted graphs. The resulting images are strongly denoised and superior in quality compared to standard filtered back-projection.

Another iterative approach aiming at improving the SNR of the reconstructed volumes, Motion Vector-based Iterative Technique (MoVIT), was proposed by Van Nieuwenhove et al*.*^16^. The algorithm uses deformation fields within the reconstruction process to exploit information available through the time sequence. The deformation field maps are estimated by applying a non-rigid registration between individually reconstructed volumes as a pre-processing step, and this information is then used during the final reconstruction procedure. The simulation and real-data applications were limited to consider a maximum of 3 adjacent time frames.

The time-interlaced model-based iterative reconstruction algorithm (TIMBIR)^17^ reconstructs dynamic objects by exploiting an interlaced view sampling scheme during data acquisition together with a 4D model-based iterative reconstruction algorithm. The approach aims at modelling the measurement noise, detector non-idealities and spatial–temporal correlations of the 4D object and has shown high reconstruction quality with decreased motion artefacts, especially when the sample is changing during each tomogram and interlaced data sampling is applied.

Myers et al*.*^18–20^ proposed a reconstruction algorithm combining elements from both discrete tomography and compressed sensing. The algorithm exploits simulated static projections to reconstruct the dynamic components separately and thresholds each dynamic reconstruction based on the expected SNR of the reconstruction. The work was later extended to a probability-driven algorithm based on maximum a posteriori (MAP) estimation^21,22^. Another compressed sensing-based solution, proposed by Chen et al.^23^, exploits a prior image reconstructed with a standard analytical reconstruction algorithm (filtered back-projection) from the union of interleaved dynamical datasets to constrain the iteratively reconstructed individual time frames. Nikitin et al.^24^ proposed a 4D tomographic reconstruction method to avoid reconstruction artefacts caused by sample evolvement during data acquisition. The implemented method, based on the concept of compressed sensing, decomposes dynamic datasets in the temporal domain using basis functions. The basis functions depend on the motion of the object and are selected according to the measured data. Furthermore, regularization is performed by minimizing the L1-norm for both spatial and temporal derivatives by adopting the primal–dual Chambolle–Pock algorithm^25^.

Van Eydhoven et al.^26^ introduced in 2015 an iterative reconstruction algorithm (rSIRT-PWC) designed to follow fluid flow through porous media. Time-regularization through piece-wise constant function fitting of each pixel’s attenuation value is exploited within the reconstruction to push the obtained image quality of strongly undersampled dynamic datasets and moreover, to exploit the properties of advancing fluid-air boundary within the reconstruction. The rSIRT-PWC algorithm assumes the time-varying object to consist of stationary (the solid matter) and dynamic regions (the fluid flow). The attenuation curve of a particular voxel in the dynamic region is modeled by a piecewise constant function over time, in accordance with an advancing fluid/air boundary. The algorithm allows reconstruction from substantially fewer projections per rotation without image quality loss. However, as for the other methods described above, several parameters need to be tweaked in order to push the reconstructed image quality to its maximum.

While the rSIRT-PWC algorithm offers an interesting possibility to exploit the continuity information across the available dynamic time sequence through time-regularization, it still relies, in a first step, on prior segmentation of the solid matter to separate the stationary region from the areas allowed to change through time (dynamic), which are continuously estimated and updated throughout the reconstruction process. For datasets with limited SNR, such as *operando* X-ray tomographic microscopy of fuel cells, extracting these dynamic and stationary regions from the reconstructed volumes and aligning the corresponding masks in a nearly perfect manner requires significant amount of manual labor. Moreover, as these sub-second dynamic experiments deliver as many as 10 tomograms per second for extended time periods, processing each of these large datasets prior to the algorithm application is infeasible.

To overcome these limitations, we propose a rSIRT-PWC-DIFF algorithm extended for interior tomography datasets, designed to reconstruct and segmented noisy datasets in a highly automatized manner enabling efficient scalability to large data volumes. The proposed algorithm operates on difference sinograms to separate the static and dynamic regions of the sample in a fully automatized manner. As no prior information of the static pixel location is needed, the proposed algorithm can be applied to reconstruct several datasets without manual interaction, making it ideal for cases where prior segmentation of the static features is difficult. In addition, the robustness of the time-domain function fitting step for highly noisy datasets is increased by considering small sub-regions (5 × 5 pixels) with a Gaussian weighting scheme instead of single pixels. Though the proposed algorithm protocol has been developed to overcome the current restrictions specifically in fuel cell data reconstruction, its application is not limited to fuel cells but can be applied to any dataset where evolving dynamics are present.

## Methods

### Background

#### Tomography model

The underlying tomography model is introduced for a 2D case assuming parallel beam geometry. Its extension to 3D is trivial.

The object of interest, represented on a pixel grid of $N$ pixels, is denoted by a column vector ${\varvec{x}}=\left({x}_{j}\right) \in {\mathbb{R}}^{N}$. A vector ${\varvec{p}}=\left({p}_{i}\right)\in {\mathbb{R}}^{M}$ is a collection of the log-corrected measured projection values or phase-retrieved projections of the object ${\varvec{x}}$ from all measured angular positions, typically distributed homogeneously between 0 and 180 degrees. The projection data ${\varvec{p}}$ and object ${\varvec{x}}$ are connected by ${\varvec{p}}={\varvec{W}}{\varvec{x}}$ where ${\varvec{W}}=\left({w}_{ij}\right)\in {\mathbb{R}}^{M\times N}$ is a collection of weights that models the contribution of each pixel $j$ to the projected value at index $i$.

Due to the ill-posedness of the system, it is typically infeasible to directly solve ${\varvec{x}}$ from the system of linear equations. Analytical reconstruction methods, such as filtered back-projection (FBP)^27^, are accurate when enough angular views of the object are available. Limited angular views or high sparsity in angular sampling lead instead to streak artefacts deteriorating the reconstruction quality. Iterative reconstruction algorithms address these limitations by exploiting prior information of the sought object, while minimizing the difference between the forward-projected estimated reconstruction and the measured projection data in an iterative manner.

#### Conventional SIRT

The Simultaneous Iterative Reconstruction Technique (SIRT)^28,29^ is an algebraic iterative reconstruction algorithm where the object ${\varvec{x}}$ is considered to consist of an array of unknowns, represented by algebraic equations. Starting from an initial reconstruction ${{\varvec{x}}}^{(0)}$, typically a zero vector, the SIRT algorithm updates the reconstruction at iteration $k$ by

$${{\varvec{x}}}^{\left(k\right)}= {{\varvec{x}}}^{(k-1)}+{\varvec{C}}{{\varvec{W}}}^{T}{\varvec{R}}\left({\varvec{p}}-{\varvec{W}}{{\varvec{x}}}^{\left(k-1\right)}\right),$$

where ${\varvec{R}}=\left({r}_{ij}\right)\in {\mathbb{R}}^{M\times M}$ and ${\varvec{C}}=\left({c}_{ij}\right)\in {\mathbb{R}}^{N\times N}$ are the diagonal matrices with the inverse row and column sums of ${\varvec{W}}$, respectively. The algorithm is known to converge to a solution of

$${\mathrm{argmin}}_{x }\left({\parallel {\varvec{W}}{\varvec{x}}-{\varvec{p}}\parallel }_{{\varvec{R}}}^{2}\right).$$

#### Conventional rSIRT-PWC

Van Eydhoven et al. introduced in 2015 an iterative reconstruction algorithm developed to follow in 4D dynamic fluid flow through a solid matrix^26^. The region-based SIRT with intermediate piecewise constant function estimation (rSIRT-PWC) algorithm, based on conventional SIRT, exploits prior knowledge of the dynamic and static regions of the sample to improve the spatial resolution of the reconstructions, resulting in improved spatial and temporal resolution of the experiment. The static and dynamic regions of the object are separated through masking (often requiring segmentation). The static regions are reconstructed using all available data across the complete time sequence, so to increase the sampling frequency, and a static mask is applied to extract the static pixels from the reconstructed high-quality image. Simultaneously, each of the dynamic time frames is reconstructed separately and a dynamic mask is applied to extract the respective dynamic pixels.

To further improve the dynamic reconstructions, suffering from high noise level and undersampling artefacts, the algorithm exploits piecewise constant functions (PWC) to model each single dynamic pixel’s attenuation curve over the complete time sequence, so incorporating time regularization within the reconstruction procedure. Through the PWC fitting, noise is effectively suppressed and the reconstruction accuracy of the dynamic region is significantly improved^26^. These dynamic and static reconstructions are finally merged for each time frame, obtaining a full 4D reconstruction of the dynamic object in high-quality.

In addition to masks, iteration parameters have to be manually selected. This algorithm foresees nested loops of rSIRT reconstructions and PWC time-regularization steps. It is therefore necessary to select the total number of iterations as well as the number of rSIRT iterations performed prior to the first PWC fitting and the number of rSIRT iterations performed between each of the following PWC steps in addition to the total number of PWC steps. Depending on the sample, tweaking these parameters can have a significant impact on the reconstruction quality while it is typically difficult to know a priori which set of parameters would lead to the optimal reconstruction.

### rSIRT-PWC-DIFF

While the conventional rSIRT-PWC algorithm has shown superior reconstruction performance on a neutron tomography dataset compared to the standard analytical reconstruction (FBP), the requirement of static and dynamic masks is a major limiting factor for datasets for which such masks are difficult and/or time-consuming to obtain. In addition, several iteration parameters need to be specified prior to reconstruction. In case of many in situ time-resolved experiments as fuel cell imaging, an adaptation to accommodate interior tomography datasets is also necessary. To overcome these limitations, an adapted rSIRT-PWC-DIFF algorithm was developed. A detailed description of the proposed algorithm is presented in the following sub-sections.

#### Difference sinogram approach

In the proposed rSIRT-PWC-DIFF algorithm, dynamic and static masks are no longer required, as the dynamic and static pixels are separated through an automated sinogram subtraction step. If a static scan or a static time frame of the object is available, the corresponding static sinogram ${S}_{S}$ can be subtracted from the dynamic sinogram ${{S}_{D}}_{n}$ for each time step $n,$ as

$${{S}_{Diff}}_{n}={{S}_{D}}_{n}-{S}_{S}$$

The resulting difference sinograms ${{S}_{Diff}}_{n}$ contain only dynamic changes and noise for each time frame $n$ and can be further used in the rSIRT-PWC-DIFF algorithm to reconstruct dynamic changes for each time frame $n$. Simultaneously, the dry sinogram ${S}_{S}$ is considered to reconstruct the static regions of the object. If the number of angular views between the static sinogram ${S}_{S}$ and the dynamic sinograms ${{S}_{D}}_{n}$ is not equal, interpolation can be applied to the sinograms prior the subtraction step.

#### Sinogram alignment

Ideally, angular information for each projection image is available and can be directly used to align the static and dynamic sinograms prior to the subtraction step. To generalize the proposed algorithm for datasets without angular information, a cross-correlation-based sinogram alignment step was implemented. In the sinogram alignment step, for each time frame $n$ maximum cross-correlation^30^ $\left(*\right)$ between the dynamic sinogram ${{S}_{D}}_{n}$ and the static sinogram ${S}_{S}$ is computed as

$$\max \left( {\left( {S_{S} {*}S_{Dn} } \right)\left[ t \right]} \right) = {\max}(\mathop \sum \limits_{d} S_{S} \left[ d \right]{ }S_{Dn} \left[ {d + t} \right])$$

The maximum correlation position is used to shift the dynamic sinograms ${{S}_{D}}_{n}$ so to align them with respect to the static sinogram ${S}_{S}$. This alignment process is fully automatized within the proposed algorithm. If the angular information is available, it can be applied directly to align the sinograms.

The cross-correlation step identifies the main structural similarities between the sinograms from the superimposed data and among the tested registration approaches produces the best alignment between the sinograms. For other types of samples, alternative registration methods based for instance on mutual information^31^ could also potentially be exploited. Any possible mismatch between the static structures of the dry and dynamic datasets, for example due to membrane swelling (“Effects of membrane swelling” section), translates as artefacts in the subtracted sinograms. When a standard analytical reconstruction algorithm, such as FBP, is applied to reconstruct these difference sinograms, misalignment artefacts become visible in the reconstructed images. However, when an iterative reconstruction algorithm, such as SIRT, is considered instead, these misalignment artefacts are partly suppressed during the iterative forward- and back-propagation steps and so, not significant in the reconstructed images.

#### Virtual sinogram for interior tomography

To increase the versatility of the proposed algorithm, further developments were made to extend it to interior tomography (INT) problems. In INT, the object support is fully or partly outside of the field of view (FOV) of the detector. This geometry often occurs in medical imaging, material science and biology applications where high spatial resolution information of a smaller region of interest (ROI) is required. If INT projections are reconstructed with standard iterative or analytical algorithms without special consideration, the reconstructions will suffer from strong artefacts^32,33^, compromising any further analysis of the reconstructed volume. To minimize these artefacts, a fully automatized virtual sinogram step^34^ was incorporated within the reconstruction algorithm.

In the virtual sinogram step, the INT sinogram is first edge-padded and a standard FBP reconstruction is performed. The pixels outside of the resolution circle (the largest circle fitting into the reconstruction grid) are then suppressed to zero to create an artificial object boundary. This modified object, now possessing an artificial compact support, is further forward projected to create a virtual sinogram, simulating now a standard sinogram where the object and its boundary are completely within the FOV. This virtual sinogram, further edge-padded, can then be used for iterative reconstruction.

#### Curve fitting on sub-regions

The curve fitting (PWC) part of the conventional approach was further developed to improve its robustness for highly noisy and slightly misaligned datasets. Instead of the gray level value of single pixels, the 2D Gaussian weighted average value of small sub-regions of 5 × 5 pixels was used to emphasize the direct neighborhood of each pixel during the fitting procedure. The image borders were padded prior to weighting. While the sub-region size can be adjusted, it is important to note that larger regions may lead to reduction in size or vanishing of small structures. For datasets with a very limited SNR, this compromise might however be necessary for successful curve fitting and further data processing. Thanks to the Gaussian weighting scheme, in this work, the used 5 × 5 pixel sub-regions did though not negatively impact the spatial resolution.

#### Automatic stopping criterion

An automatic stopping criterion for selecting the optimal number of iterations was defined. For this purpose, the proposed algorithm was first run for 700 rSIRT-DIFF iterations without applying the function fitting step. After every 10 iterations, the current reconstruction estimate was compared to the reconstruction obtained 10 iterations earlier through the Euclidean L2-norm, defined for images **x** and **y** as

$$L2\left(\mathbf{x},\mathbf{y}\right)= \sqrt{{(\mathbf{x}-\mathbf{y})}^{2}}.$$

**Figure 1:**
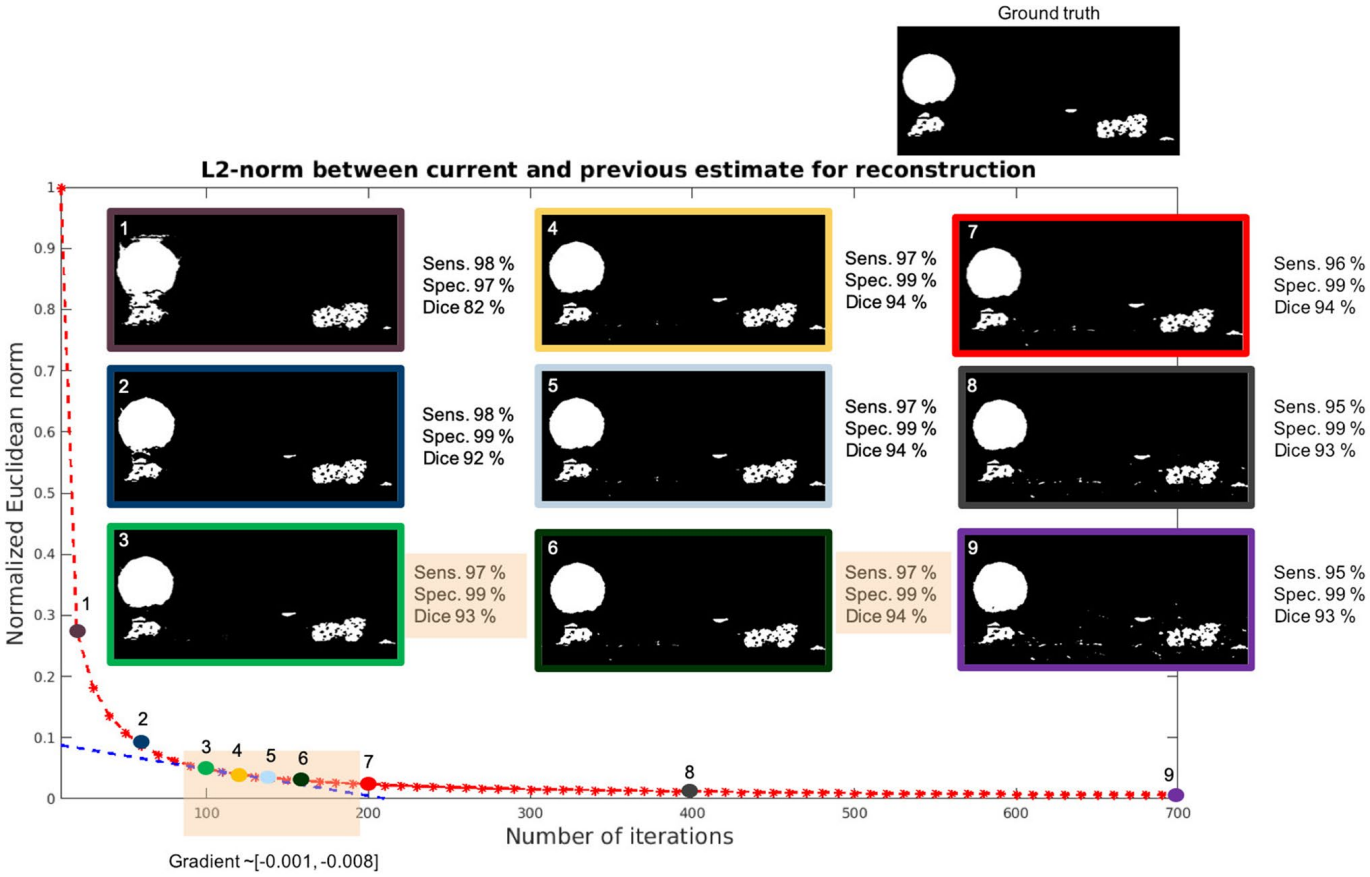
Automatic stopping criterion evaluation. Euclidean L2-norm (red curve) between the current and previous reconstruction estimate of PEFC_1 sample (“Real data” in “Materials” section) was measured after every 10 iterations for a total of 700 iterations. The gradient of the L2-curve was estimated and reconstruction quality measured at selected gradient slope positions. The gradient slope (blue curve) within region $\left[- 0.001,- 0.008\right]$ was identified to correspond to the highest reachable reconstruction quality. The droplet diameter is 100 pixels.

The normalized L2-norm was plotted as a function of the number of iterations and its gradient was calculated. Several gradient slopes were selected for further analysis (Fig. 1) and the corresponding reconstructions were post-processed by applying a single PWC fitting step. The obtained reconstructed dynamics (here water) were compared to a ground truth segmentation through sensitivity (true-positive-rate), specificity (true-negative-rate)^35^ and the dice coefficient^36^. The metrics are defined as:

$$\text{Sensitivity} = \frac{\text{TP}}{\text{TP} + \text{FN}}$$

$$\mathrm{Specificity}=\frac{\mathrm{TN}}{\mathrm{TN}+\mathrm{FP}}$$

$$\mathrm{Dice}=\frac{2\times \mathrm{TP}}{2\times \mathrm{TP}+\mathrm{FP}+\mathrm{FN}}$$

where TP are true-positive pixels (water reconstructed as water), FP are false-positive pixels (air reconstructed as water), TN are true-negative pixels (air reconstructed as air) and FN are false-negative pixels (water reconstructed as air). Based on quantitative and qualitative analysis (Fig. 1) on both simulated (“Simulated phantom” section) and real data (“Real data” section), a gradient slope within a region of $[-0.001,-0.008]$ was identified as an optimal stopping point for the rSIRT-DIFF iterations and starting point for the PWC step. In this gradient slope region, the reconstructions recover the sample features successfully without emphasizing noise, leading to a desirable starting point for the time-regularization. Moreover, within this gradient slope region the reconstruction quality remained stable indicating only a low dependence on the iteration number around its optimum. If the iteration number is not high enough, the water features are instead not properly defined. Too many iterations lead to noisy reconstructions. Additional rSIRT-DIFF/PWC fitting step loops as proposed in^26^ did not improve the reconstruction quality. In addition to the higher computational load, more loops tend to increase the level of noise in the reconstructed volumes. The described analysis was conducted on a simulated fuel cell phantom with high and moderate noise levels (“Simulated phantom” section) and on two real fuel cell datasets (“Real data” section). For all considered cases, the normalized L2-curve was behaving in a similar manner, only its relative position was shifted in y-direction. In all cases the optimal stopping point was identified to correspond to a gradient slope of $\left[-0.001,-0.008\right]$, which consistently with Fig. 1 was reached with 100–200 iterations. The same optimal gradient slope, obtained for both simulated and real fuel cell data, was found to be independent on image size and contrast. Therefore, for all fuel cell samples the number of rSIRT-DIFF iterations was set, to maximize computational performance, to 100, followed by a single PWC fitting step. For completely different types of samples (for instance sandstone or catalytic materials), the optimal value for the gradient slope has to be confirmed.

#### Algorithm protocol

**Figure 2:**
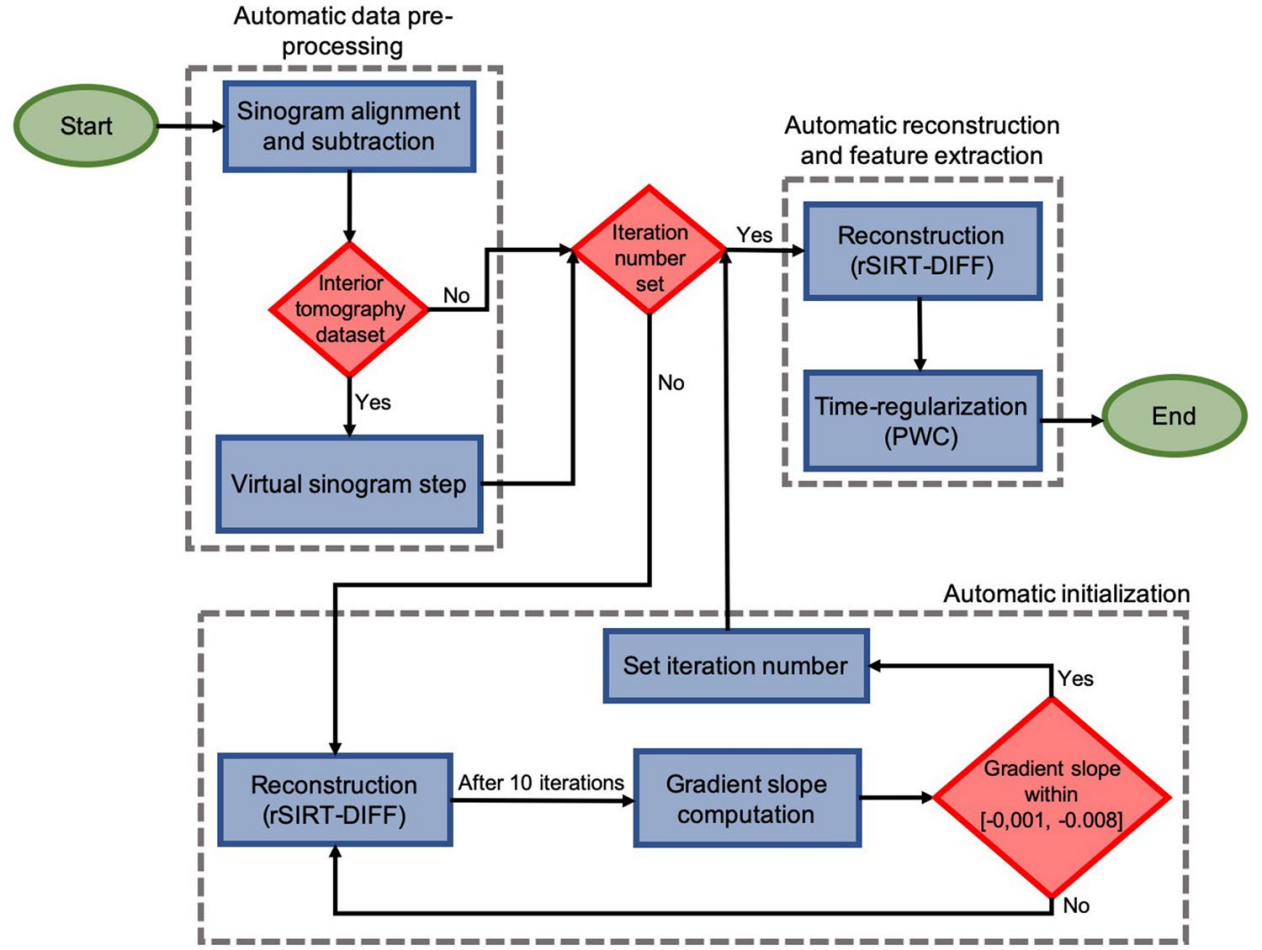
Algorithm protocol flowchart. The algorithm protocol begins by automatically pre-processing the sinograms to extract the dynamic components. If the required number of iterations is not yet set for the current sample type, the protocol moves automatically to the “Automatic initialization” module. Once the number of required iterations has been determined, the pipeline proceeds automatically to the “Automatic reconstruction and feature extraction” step. The set iteration number will be directly re-used for other datasets of similar sample types: the “Automatic initialization” step will be skipped and the protocol will automatically transition from the pre-processing to the reconstruction step.

To apply the proposed rSIRT-PWC-DIFF algorithm, we propose the following protocol described in Fig. 2:

1. Align and subtract sinograms to extract the dynamic changes (“Sinogram alignment” section).
2. If the dataset was acquired in interior tomography geometry, apply the virtual sinogram step (“Virtual sinogram for interior tomography” section).
3. Automatic initialization: For each new sample type, run rSIRT-DIFF once for a large number of iterations (suggested 700). Automatically stop iterations when the gradient slope of the L2-curve reaches the interval [− 0.001, − 0.008] (“Automatic stopping criterion” section).
4. Reconstruct with the rSIRT-PWC-DIFF algorithm:
  - Run rSIRT-DIFF for the number of iterations based on the gradient slope estimation (step 3).
  - Apply one iteration of time-regularization (PWC) to 5 × 5 pixel sub-regions of the dynamic reconstruction.

Step 3 has to be performed once for each sample type to determine the desired number of iterations corresponding to the specified gradient slope region. Once the stopping point has been automatically detected, the algorithm can be further applied in a faster manner by directly applying the chosen number of iterations without estimating the L2-norm. If the number of rSIRT-DIFF iterations specified by the gradient slope is used, additional loops of reconstruction and time-regularization steps were not found to improve the reconstruction quality further. Therefore, for optimized performance and computational load, one single cycle of reconstruction and time-regularization is recommended.

## Results

In this section, reconstruction results of the rSIRT-PWC-DIFF algorithm are presented for a simulated fuel cell phantom (“Simulated phantom” section) and two dynamic X-ray tomographic microscopy datasets of a fuel cell (“Real data” section). The reconstructions were completed with MATLAB (2018b) using the ASTRA toolbox^37–39^ and exploiting a Tesla V100 GPU card for accelerated computations.

### Simulated phantom

#### Standard setting

**Figure 3:**
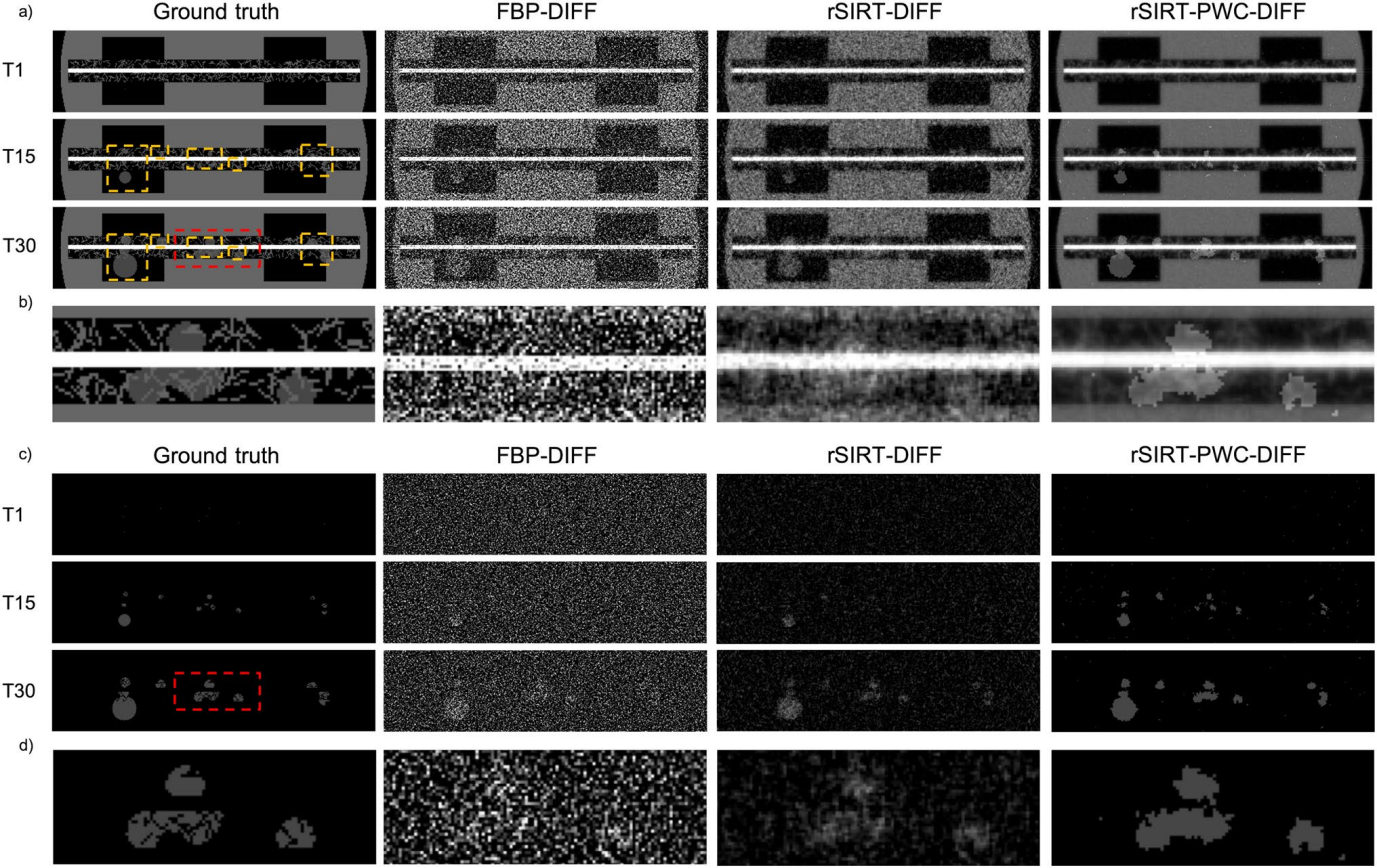
Reconstruction results for a simulated dynamic phantom. The ground truth phantom (first column) is shown at time frames 1, 15 and 30, cropped to the region-of-interest. The water droplets (highlighted by orange rectangles in (**a**) in the gas channel and gas diffusion layers are evolving in time. The corresponding FBP-DIFF (second column), rSIRT-DIFF (third column) and rSIRT-PWC-DIFF reconstructions (fourth column) are considered for comparison. (**a**) Reconstructions are presented for time frames 1 (first row), 15 (second row) and 30 (third row). All reconstructions were completed using difference sinograms to separate the dynamic (water) structures from the static areas. Separate dynamic and static reconstructions were merged to obtain the presented reconstructions. (**b**) Zooms in a reconstructed region at time step 30. (**c**) Reconstructions of the dynamic (water) structures, prior merging with the reconstruction of the static matrix. (**d**) Magnification of reconstructed dynamic (water) structures at time step 30.

To test and quantify the developed algorithm performance, a dedicated fuel cell phantom was created (Fig. 3, Section “Simulations” in “Materials”). The difference between the ground truth phantom and the reconstructions was estimated by the relative root mean squared error (RRMSE), defined as,

$$RRMSE\left( {x} \right) = \sqrt {\frac{{\mathop \sum \nolimits_{i} \left( {x\left( i \right) - y\left( i \right)} \right)^{2} }}{{\mathop \sum \nolimits_{i} \left( {y\left( i \right)} \right)^{2} }}}$$

where $x$ is the reconstructed image, $y$ the ground truth phantom and $i$ the time frame.

Reconstructions obtained with the rSIRT-PWC-DIFF algorithm were compared to standard FBP (Parzen filter) and rSIRT reconstructions of a difference sinogram (FBP-DIFF and rSIRT-DIFF respectively) without the function fitting step. For rSIRT-DIFF and rSIRT-PWC-DIFF, a positivity constraint was enforced by setting negative pixel values to zero during the iterative process. For both iterative methods, a total of 100 iterations was considered based on the defined stopping criterion (“Automatic stopping criterion” section), followed by a single PWC-fitting for rSIRT-PWC-DIFF. The RRMSE metric was measured within three regions: full region-of-interest (ROI), static ROI and dynamic ROI, selected by applying corresponding masks. Results for all three reconstruction methods are presented in Table 1.

**Table 1:** Average relative root mean squared error (RRMSE) estimates and their standard deviations for the dynamic fuel cell phantom reconstructions computed using a difference sinogram with FBP (FBP-DIFF), rSIRT (rSIRT-DIFF) and rSIRT-PWC-DIFF. The error metric was computed for the full ROI, the static ROI and the dynamic ROI. Static and dynamic ROIs were separated for evaluation using corresponding masks. The metrics were calculated for 100 fuel cell phantoms, each having randomized fiber and water content.

	FBP-DIFF	rSIRT-DIFF	rSIRT-PWC-DIFF
Full ROI	1.21 ± 7.3E−4	0.32 ± 5.1E−4	0.18 ± 7.8E−4
Static ROI	1.15 ± 6.3E−4	0.29 ± 2.1E−4	0.13 ± 4.2E−4
Dynamic ROI	3.04 ± 0.05	1.11 ± 0.02	0.98 ± 0.01

The error measures in Table 1 show superior reconstruction performance for all three ROIs when the rSIRT-PWC-DIFF algorithm was applied. In comparison to FBP-DIFF, the proposed algorithm improves the reconstruction of the full ROI by a factor of 6.7. The quality in the static ROI was improved by a factor of 8.8 while in the dynamic ROI by a factor of 3.1. Comparing the results for rSIRT-DIFF and rSIRT-PWC-DIFF, it is evident that including the PWC step improves the reconstruction quality in all three ROIs, and in particular of a factor of 1.13 in the dynamic (water) region. These improvements are also clearly visible in Fig. 3, presenting an example of the reconstructions obtained with the three algorithms and ground truth phantom for the merged and dynamic-only reconstructions. Increased sharpness in the static, fibrous structures (Fig. 3a,b) could be achieved by considering a high-resolution static scan, as typically done in fuel cell imaging experiments (“Real data” in “Materials” section).

#### Effects of membrane swelling

During fuel cell operation, the hydration of the polymer electrolyte membrane can change, which leads to swelling or shrinkage of the membrane, such that the corresponding bright area within the cell center (Fig. 3) changes its thickness accordingly. Due to this swelling, all dry structures within the GDLs are repositioned by the increasing membrane layer size, causing a mismatch between the dry structures of the dry and wet (dynamic) scans. Typically, 1 to 2 pixels (2.75–5.5 µm) relative shift between some of the dry and wet scans’ dry structures can be observed in X-ray tomographic volumes.

**Figure 4:**
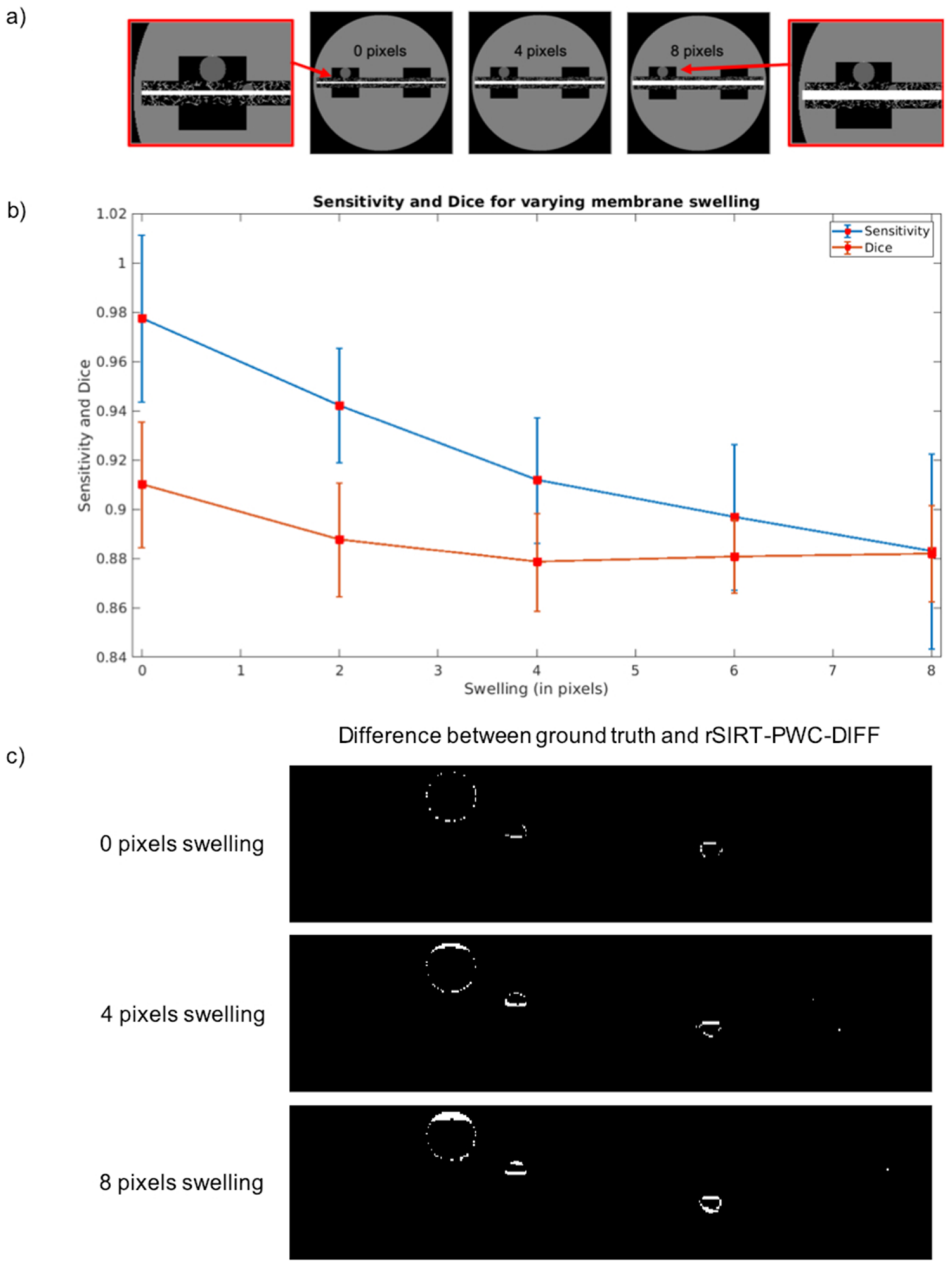
Membrane swelling effect assessment in a simulated dynamic phantom. (**a**) The dynamic fuel cell phantom at time frame 30 with simulated membrane swelling. The increased volume of the platinum layer (bright rectangular area) pushes the static fibrous structures, causing a mismatch in the dry structure positions between the dry (no swelling) and dynamic (swelling) simulated data. (**b**) Sensitivity and dice coefficient of the rSIRT-PWC-DIFF reconstruction of water versus membrane swelling size in pixels. The metrics and their standard deviations were considered between the ground truth phantom and the rSIRT-PWC-DIFF reconstruction for time steps 10 to 30, completed with a total of 100 rSIRT-DIFF iterations, followed by a single PWC fitting. (**c**) Difference image between the dynamic rSIRT-PWC-DIFF reconstruction and ground truth phantom of water at time frame 30. When no swelling is applied (top), slight reconstruction errors are visible at the water boundaries. These errors get more pronounced with increased swelling (middle and bottom) due to shifting dry boundaries.

To quantify the effects of membrane swelling on the rSIRT-PWC-DIFF reconstruction quality, membrane swelling of 0 to 8 pixels was simulated (Fig. 4a) for the dynamic fuel cell phantom. The water reconstruction accuracy was quantified through sensitivity (true-positive-rate), specificity (true-negative-rate) and the dice coefficient (Section Automatic stopping criterion). The rSIRT-PWC-DIFF reconstructions were obtained by completing 100 iterations of rSIRT-DIFF, chosen based on the stopping criterion (Section Automatic stopping criterion) and followed by a single PWC fitting step. The reconstruction quality was evaluated by comparing the dynamic reconstructions (water) without merging the static structures to the ground truth dynamic phantom (water only) between time steps 10 and 30. Results are presented in Fig. 4b,c.

As demonstrated in Fig. 4b, the sensitivity of the reconstruction remains at 90% or above for mild swelling of maximum 6 pixels, decreasing with increasing swelling. This is also supported by qualitative analysis: Fig. 4c reveals that increasing the applied swelling translates to amplified differences between the reconstructed and ground truth phantom as the misalignment artefacts of the moving static structure boundaries become visible and pronounced, leading to decreased reconstruction accuracy. These artefacts could be partially suppressed by aligning the dry scan independently for the anode and cathode sides of the cell prior subtraction, as it is typically done in practice. The dice coefficient (Fig. 4b) was found to remain between 91 and 88% for all applied swellings. The specificity was found to remain stable at 99% for all considered swelling scenarios.

### Real data

The proposed algorithm performance was evaluated on two dynamic Polymer Electrolyte Fuel Cell (PEFC) synchrotron tomographic datasets described in the “Materials” section. For PEFC_1, the rSIRT-PWC-DIFF reconstructions were computed for a time sequence of 30 consecutive time frames and for PEFC_2 a total of 10 consecutive time frames were considered, based on pre-evaluation of the water dynamics in the data and availability of manual segmentations for quality comparison. The number of iterations was chosen based on the stopping criterion (“Automatic stopping criterion” section), resulting in 100 rSIRT-DIFF followed by a single PWC fitting step with 5 × 5 pixel sub-regions.

#### Reconstruction results

**Figure 5:**
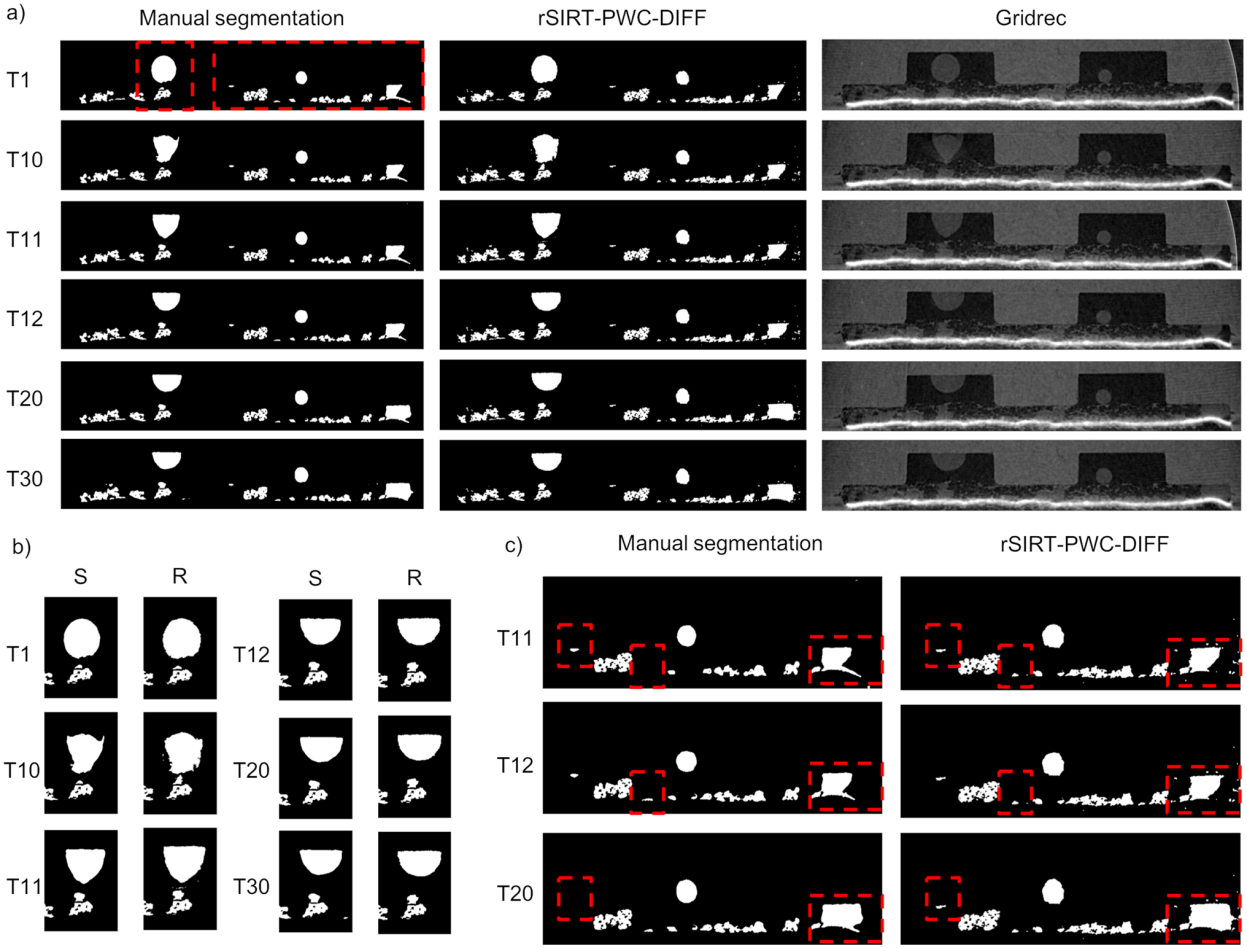
Reconstruction results for the PEFC_1 dataset. (**a**) Comparison of the recovered water from a fuel cell sample (PEFC_1) between manual segmentations (left column) and rSIRT-PWC-DIFF reconstructions (middle column). The manual segmentations have been created from standard Gridrec reconstructions (right column) by applying an ad hoc post-processing pipeline^12^. Each row represents a single time step from the continuous time sequence of a total of 30 time frames. (**b**) Zoom to the large droplet evolving strongly between time steps 10 and 12. The left columns (S) correspond to the manual segmentations, the right columns (R) to the rSIRT-PWC-DIFF reconstructions. (**c**) Zoom to time steps 11, 12 and 20, revealing small structures disappearing and re-appearing in the manual segmentations (left column). The same features were found to remain stable in the rSIRT-PWC-DIFF reconstructions (right column). The misalignment artefacts in the manual segmentations (bright curve) are suppressed in the iterative reconstructions.

The rSIRT-PWC-DIFF reconstructions of the PEFC_1 are presented in Fig. 5a (middle column) for time frames 1, 10, 11, 12, 20 and 30, cropped to the area containing the dynamic changes. The reconstructions are compared to the manual segmentations (left column) and standard Gridrec reconstructions (right column). For both samples, the manual segmentations were generated by aligning the dynamic Gridrec reconstructions to an additional high-quality dry Gridrec reconstruction of the sample (1000 projections with 1 ms exposure time) followed by an image subtraction step, resulting in images containing only dynamic changes (water) and noise^11^. These subtracted images were further post-processed to generate the final segmentations for each time frame^12^. In addition, for PEFC_1 a static mask containing GDL fiber structures has been applied to the manually segmented images as a post-processing step to exclude any fiber structures misclassified as water. For comparison, the same mask was applied as a post-processing step to the rSIRT-PWC-DIFF reconstructed images. The reconstructions were evaluated through sensitivity, specificity and dice coefficient measures (“Automatic stopping criterion” section), results are presented in Table 2.

**Table 2:** Sensitivity, specificity and dice coefficient between the rSIRT-PWC-DIFF reconstructions and manual segmentations for PEFC_1 and PEFC_2 datasets. For PEFC_1 each metric was computed over the whole image plane and averaged over the time sequence. For PEFC_2 the metrics were averaged over the time sequence at chosen sub-regions due to remaining misalignment artefacts in the segmented images (Fig. 6a).

Dataset	Sensitivity	Specificity	Dice
PEFC_1	0.97	0.99	0.94
PEFC_2	0.92	0.98	0.93

As revealed by Fig. 5a, the rSIRT-PWC-DIFF reconstructions are visually in good agreement with the manual segmentations. The dynamic changes, most rapid at time steps 10 to 12 (Fig. 5b), are well captured while slight misalignment artefacts in the manual segmentations (bright curve at the right side of Fig. 5c) are successfully suppressed in the rSIRT-PWC-DIFF reconstructions. Moreover, Fig. 5c reveals small water structures disappearing and re-appearing in the manual segmentations, while the structures remain stable in the rSIRT-PWC-DIFF reconstructions. This qualitative performance is supported by the error metrics in Table 2: high sensitivity of 97% is reached across the time sequence while the specificity is 99% and the dice coefficient 94%. Even slightly higher sensitivity and dice coefficient can be expected if manual segmentations are post-processed to exclude any slight misalignment artefacts.

**Figure 6:**
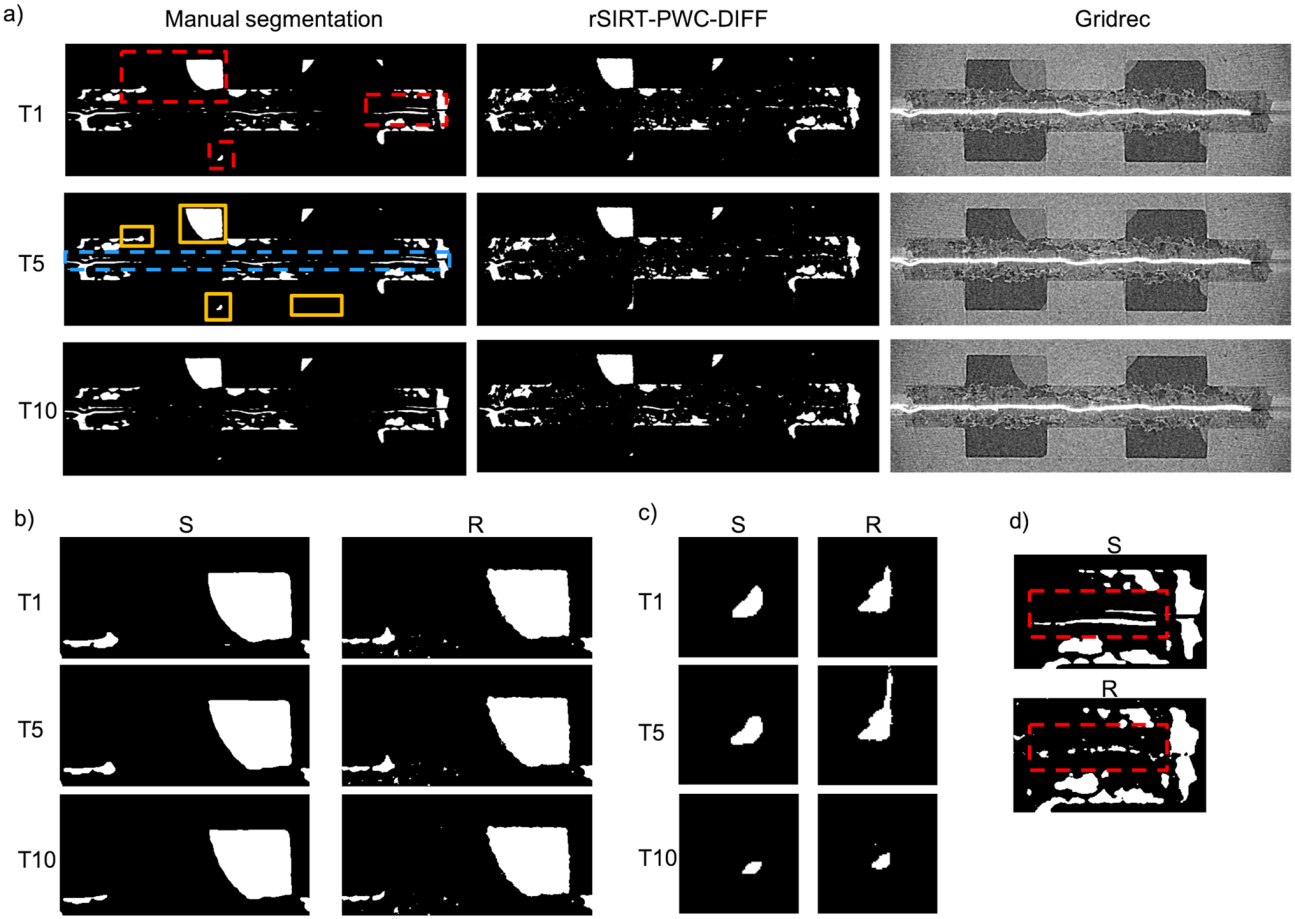
Reconstruction results for the PEFC_2 dataset. (**a**) Comparison of the recovered water from a fuel cell sample (PEFC_2) between manual segmentations (left column) and rSIRT-PWC-DIFF reconstructions (middle column). The manual segmentations have been created from standard Gridrec reconstructions (right column) by applying an ad hoc post-processing pipeline^12^. Each row represents a single time step from the continuous time sequence of a total of 10 time frames. The manual segmentation suffers from misalignment artefacts (blue dashed rectangle at T5). To avoid considering these artefacts during accuracy evaluation, the reconstruction quality measures (sensitivity, specificity and dice coefficient) were computed only for chosen sub-regions (yellow rectangular areas), for which the segmentation was considered accurate based on visual comparison with the original Gridrec reconstructions. (**b**–**d**) Zoom to the evolving structures indicated by red rectangles in (**a**). (S) panels correspond to manual segmentations, (R) panels to the rSIRT-PWC-DIFF reconstructions. The misalignment artefacts in the manual segmentations (S) (red rectangle in (**d**)) are suppressed in the iterative reconstructions (R).

For the PEFC_2 sample, the rSIRT-PWC-DIFF reconstructions are presented in Fig. 6a (middle column) for time frames 1, 5 and 10, cropped to the area containing the dynamic changes. For comparison, the manual segmentations (left column) and standard Gridrec reconstructions (right column) are presented. For the manual segmentations of PEFC_2 no additional post-processing step to exclude fibers has been considered and so, this step has been omitted for the rSIRT-PWC-DIFF reconstructions as well. The manual segmentations were found to suffer from misalignment artefacts (Fig. 6a) due to sub-optimal alignment of the dry and dynamic reconstructions prior the subtraction step. To avoid evaluating such artefacts in the iterative reconstructions, the reconstruction quality was measured only in chosen sub-regions (Fig. 6a) for which the segmentations could be visually confirmed successful based on the original Gridrec reconstructions. The sensitivity, specificity and dice coefficient measures (“Automatic stopping criterion” section) are reported in Table 2.

Figure 6 shows high agreement between the manual segmentations and the rSIRT-PWC-DIFF reconstructions. While the dynamic process was found to proceed at moderate speed, the slight changes through the time sequence (Fig. 6b,c) are captured well while the stable water features are correctly reconstructed and remain static through the time sequence. Moreover, the misalignment artefacts (Fig. 6a) are found to be suppressed in the rSIRT-PWC-DIFF reconstructions (Fig. 6d). The qualitative comparison is supported by the quality metrics considered at chosen sub-regions (Fig. 6a) and presented in Table 2: the sensitivity of the reconstructions across the time sequence was 92%, with high specificity of 98% and dice coefficient of 93%.

## Discussion

We have introduced the rSIRT-PWC-DIFF algorithm, designed to reconstruct and segment, in an unsupervised manner, dynamic, low SNR interior tomography datasets consisting of static and dynamic structures without prior knowledge of the sample composition and its inner features. The proposed algorithm exploits difference sinograms between static and dynamic tomograms to directly extract dynamic features automatically without need for prior reconstruction and segmentation. Time regularization in terms of a piecewise constant function fitting (PWC) approach is applied to 5 × 5 pixel sub-regions with a Gaussian weighting scheme, so to effectively suppress noise within the dynamic reconstructions. Moreover, a stopping criterion was implemented to determine automatically the number of required rSIRT iterations and the starting point of the time-regularization step. Strive for automatization has been guiding this work: prior knowledge and input required by the algorithm has been strongly minimized. In this way, the algorithm can be applied unsupervised to large datasets with hundreds of time steps and to datasets of different samples.

Currently the algorithm works on single slices independently assuming that the sample is stable in the vertical direction, while drifts in the axial plane are automatically accounted for. To generalize the approach further, it is necessary in the future to include sinogram alignment in 3D. In this way any arbitrary drift in the vertical direction, not uncommon in evolving samples, could also be corrected during the alignment process, so to minimize any misalignment artefacts.

The current sinogram alignment is sufficient for aligning scans for which the relative position of the sample with respect to the rotation axis has remained stable. However, in cases where the sample position relative to the rotation center has changed significantly between scans, the current sample alignment will result in reconstruction artifacts arising from disagreeing sinogram shapes. In such cases we recommend to perform the current alignment procedure on a selection of lines (batches), the selection size depending on the amplitude of the relative sample position change, so to achieve an optimized alignment. Alternatively, an additional pre-processing step can be performed, in which the scans are first reconstructed (e.g. with FBP), coarsely aligned and after this, forward projected for final alignment followed by subtraction and the proposed iterative reconstruction. By default, the approach aligns sinograms assuming stable relative sample position for faster computation.

Currently the reconstruction, exploiting a GPU for the rSIRT iterations, is performed in approximately 2.5 min for one slice with 30 time steps (PEFC_1 dataset). To expand the possible applications for large-scale datasets, improvements in computational efficiency are foreseen. For accelerated reconstruction, an interesting possibility is to exploit approximated algebraic filters^40–42^ to obtain reconstructions with comparable image quality to SIRT with the computational effort of FBP. Moreover, further improvement of the efficiency of the time-regularization step is envisaged through neural networks which have already demonstrated impressive results in improving the tomographic reconstruction quality when used as a post-processing tool^43,44^.

For datasets with highly limited SNR and complicated sample structures, common in time-resolved X-ray tomographic microscopy investigations, the typical data processing pipeline includes data reconstruction, registration, filtering and segmentation, each step requiring manual parameter tuning and optimization. Once the optimal parameters have been identified, the pipeline can be typically applied to a time sequence with only minor parameter tweaking and manual intervention. Unfortunately, the parameters do though not generalize well, requiring a new parameter evaluation when experimental conditions or samples are modified. These major manual requirements considerably extend the total data post-processing time, limiting possible dynamic analysis realistically to a few samples and time steps. The proposed algorithm protocol offers a significant reduction in manual labor while delivering robust and high-quality reconstructions. This was demonstrated by automatically extracting liquid water dynamics from sub-second fuel cell X-ray tomographic microscopy datasets. The post-processing pipeline used so far to extract water dynamics from reconstructed images relies on subtracting a dry scan from dynamic scans. Manual tuning and supervision are unavoidable to optimally adjust the scan alignment and filtering parameters for these SNR-limited data to obtain segmented water. The parameters are dataset specific and need to be adjusted for each experiment individually. Despite careful alignment, remaining misalignment artefacts are inevitable, leading to the need for careful post-processing to avoid such artefacts appearing as falsely detected water in the final segmented images. For good quality data of a single cell, considering 100 full volume time steps, the complete process from raw data to segmented water requires in the best case 2 weeks of work, significantly dominated by the necessary manual interaction and not by the computation distributed on a maximum of 15 CPU nodes. In case of challenging image quality or completely new cell types and materials, additional manual tuning and exploration of the parameter space might be necessary, extending the processing time from 2 weeks up to 1 month. Thanks to the generalization of the stopping criterion, the proposed algorithm enables to perform reconstruction and segmentation of comparable data fully automatically in 1 week on a single GPU node. In addition, thanks to the subtraction step performed prior to back-projection and time-regularization, misalignment artefacts are optimally suppressed. As the algorithm is completely independent from manual intervention, it can be easily scaled up to efficiently process even larger data amounts by utilizing additional GPUs and parallelization. The reduced total post-processing time as well as the fully automatized protocol will open up new possibilities in dynamic X-ray investigations (e.g. exploration of a considerably larger experimental parameter space), for which the manual effort required by conventional pipelines would represent an unsurmountable obstacle. Though this algorithm has been designed to meet especially the needs and requirements of fuel cell reconstruction, it is not limited solely to fuel cell imaging applications but can be applied to any dynamic dataset for which either a static reference scan or time frame is available. Thanks to the high-quality reconstructions of limited SNR datasets delivered by the proposed rSIRT-PWC-DIFF in a highly automatized manner, it will be possible to push the temporal resolution of dynamic tomography experiments even further to the sub-second scan time region and beyond and efficiently deal with the related large data volumes.

## Materials

### Simulations

The fuel cell phantom was designed to mimic the static structures of an operando X-ray tomographic microscopy fuel cell setup^45^: a flow field with two gas channels was placed both on the anode and cathode sides of the cell together with a gas diffusion layer (GDL) consisting of thin, randomly distributed carbon fibers. A bright layer was added between the anode and cathode GDLs to mimic the polymer electrolyte membrane coated with Pt-based catalyst. A total of 100 phantoms were simulated with randomized GDL fiber structure on a 400 × 400 pixel grid, each having one larger droplet randomly positioned in one of the channels. In addition, 2 to 20 smaller droplets were randomly chosen and positioned within the GDL. All simulated droplets were evolving in time.

Poisson distributed noise, assuming an incoming beam intensity of 5 × 10^3^ photons per pixel, was applied to the standard simulated data to simulate strongly noisy images and to avoid algorithm overfitting. The estimated SNR of the simulated FBP reconstructions was a factor of 3.7 lower than the SNR of the phase retrieved Gridrec reconstructions of the real data (“Real data” section), leading to a performance assessment for strongly noisy imaging conditions.

The membrane swelling was simulated by shifting all dry and dynamic structures in the wet simulated scans in vertical direction while the dry scan phantom remained unchanged. To ensure that most misclassifications are related to misalignment, a reduced Poisson distributed noise, compared to the previous experiment was applied. The chosen incoming beam intensity of 30 × 10^3^ photons per pixel lead to a comparable SNR in a simulated FBP reconstruction as measured in phase retrieved Gridrec reconstruction of real data (“Real data” section).

### Real data

Two dynamic Polymer Electrolyte Fuel Cell (PEFC) synchrotron tomographic datasets were collected at the beamline for TOmographic Microscopy and Coherent rAdiology experimenTs (TOMCAT) at the Swiss Light Source (SLS) at the Paul Scherrer Institut, Switzerland^46^. Both PEFCs had a diameter of approximately 5 mm and were mounted on the rotation stage. For both experiments, a high-numerical-aperture macroscope^47^ providing a 4 × magnification was coupled with a 150 µm thick LuAG:Ce scintillator (Crytur, Turnov, Czech Republic) to convert the X-rays into visible light. The in-house developed GigaFRoST detector^48^ was exploited to enable continuous data acquisition at the maximum rate of nearly 8 GB s^−1^. The radiograms were acquired using filtered polychromatic radiation with a peak energy of approximately 20 keV. To match the required time resolution, the horizontal field of view was reduced to approximately 4 mm, leading to interior tomography datasets.

The first cell (PEFC_1) (flow field plates made of BMA5, SGL Technologies; membrane electrode assembly: Gore Primea A510.1/M815.15/C510.4 with a 15 µm thick membrane and anode/cathode Pt loadings of 0.1/0.4 mg/cm^2^; GDL: Toray TGP-H-060, Toray Industries Inc.) was connected from above with two long tubes which provided the cell with continuous gas flows, enabling continuous cell rotation during operation. To avoid blurring artefacts caused by continuous sample rotation during data acquisition, 300 evenly distributed projections of the PEFC_1 sample were collected between 0 and 180 degrees. The exposure time was set to 0.3 ms for each projection image, leading to a total scan time of 0.1 s per tomogram. The collected images were 1440 × 1100 pixels with a pixel size of 2.75 µm. A single dry scan was also collected with the same experimental parameters. Five sets of tomographic datasets were acquired, each consisting of 60 continuous time steps. The data for the first 3 sets (3 × 60 scans) are available in TomoBank^49^.

For the second cell (PEFC_2) (flow field plates made of BMA5, SGL Technologies; membrane electrode assembly: Gore Primea A510.1/M815.15/C510.4 with 15 µm thick membrane and anode/cathode Pt loadings of 0.1/0.4 mg/cm^2^; GDL: SGL 28BC, SGL Carbon), a heated fluid rotary union was developed to exploit continuous gas flow and sample rotation at elevated operation temperature during the experiment. A total of 120 tomograms were collected continuously, each time frame consisting of 299 homogenously distributed projections. The scan time was set to 0.1 s per tomogram, leading to an exposure time of 0.33 ms for each projection. The collected images were 1440 × 1000 pixels with a pixel size of 2.75 µm. A single dry scan was collected with the same experimental parameters.

Prior to reconstruction, all projection images were dark and flat-field corrected, followed by phase retrieval^50^ using the processing pipeline available at TOMCAT^51^. Paganin phase retrieval was applied together with a deconvolution step to enhance the contrast between different materials while aiming to maintain the spatial resolution, typically compromised by the phase retrieval procedure^52^.

## Data Availability

The PEFC_1 dataset is available in TomoBank (https://tomobank.readthedocs.io/en/latest/source/data/docs.data.dynamic.html#foam-data). The PEFC_2 dataset and the code are available upon request.
